# Plasma-discharge-integrated slot structure for microwave power limiter

**DOI:** 10.1038/s41598-023-37336-1

**Published:** 2023-06-22

**Authors:** Jeong Min Woo, Mun No Ju, Jae-Bok Lee

**Affiliations:** Electrical Environment Research Center, Korea Electrotechnology Research Institute (KERI), 12, Bulmosan-Ro 10 Beon-Gil, Changwon, 51543 South Korea

**Keywords:** Engineering, Materials science

## Abstract

A slot structure was combined with a discharge electrode to limit incident high-power microwaves via the integration of plasma discharge. At the target resonating frequency of 9.45 GHz, the surface current was concentrated at an electrode, and the electric field was enhanced by the proposed design to lower the response power level of the incident signal. When a low-power signal is injected, plasma is not generated, and the incident wave travels without insertion loss. Double-stage slot structures were utilized to broaden the band-pass characteristics in the frequency domain, and the demonstrated plasma limiter exhibited an insertion loss of 1.01 dB at 9.45 GHz. The xenon gas pressure was optimized with the shortest distance of 100 µm between the upper and lower electrodes to reduce the discharge power of the plasma. In the case of a high-power signal input, as xenon-gas breakdown occurred, the transmitted signal was close to zero, and most of the high-power signal was reflected with a blocking efficiency of 40.55 dB. The demonstrated result will be useful to protect the receiver of a radio detection and ranging system from the high power microwave.

## Introduction

High-power microwaves have attracted increasing attention in advanced communication technologies. High-power microwaves are used in the driving of plasmas in tokamaks and radio-frequency acceleration in linear colliders^1^, while in the case of military applications, they are used to detect fighters, warships, and missiles^2–4^. To realize an improved signal-to-noise ratio and a longer distance detection in the case of a radio detection and ranging (RADAR) system, the output power of the system is increased^5,6^. The basic RADAR working mechanism theory can be divided into transmission and reflection. Transmission pulses are radiated using an antenna, and electromagnetic waves are scattered from the targets encountered. A portion of the scattered signal is returned to the receiving antenna and finally processed. However, high-power electromagnetic waves generated from ground-surface reflection^7^, reflection from a nearby metal surface^8^, intentional electromagnetic interference^9^, and naturally generated lightning electromagnetic pulses (EMPs)^10–12^ may cause temporary or permanent damage to the system, especially at the receiver, because its function is to amplify a small signal^13–17^.

These EMP threats are largely categorized as front-door coupling and back-door coupling. Front-door coupling is an EMP that is coupled to the RADAR through the antenna of the communication equipment that is designed to couple the power^18,19^. Back-door coupling refers to a malfunction or destruction of equipment when an EMP is coupled to a part of the cable or the device that connects to a communication network or equipment through a structural hole or gap^20–22^. To establish this EMP protection technology, it is categorized according to the type of coupling. The RADAR system technology that protects against front-door coupling can be categorized into various types such as solid-state, ferrite, and plasma types; when they operate ideally, the insertion loss must be below a specific input power value, and the loss must be generated above a specific input power value in order to function as a protection device. Solid-state limiters and semiconductor-based protection devices consist of P-I-N diodes, Schottky diodes, and field effect transistor devices^23–26^. The most representative semiconductor limiter, which comprises a parallel P-I-N diode, has the advantage of having a low insertion loss value in a low-power input signal. The performance and operating frequency band are determined based on the characteristics of the semiconductor device. However, the device has the disadvantage of exhibiting a low reflection or absorption coefficient for a high input power^24^. The ferrite limiter exhibits nonlinear absorption according to the input microwave power^27–29^. These magnetic materials convert power that is over the critical power limit into heat in order to protect the system. The ferrite limiter exhibits a narrowband response characteristic to a high input power; this characteristic responds only to a specific frequency, which is disadvantageous because it is difficult for a user to select an operating frequency. In addition, because the system is protected through absorption rather than reflection, additional insertion loss occurs compared to that in the case of other types of limiters. The size of ferrite limiter is generally large, and the threshold power value is difficult to adjust.

The output power from the protection device must be within the operating range of an active high-frequency circuit, such as a low-noise amplifier. Accordingly, the operating frequency range, bandwidth selectivity, insertion loss, and input power limit value of the device are important evaluation values for the RADAR/antenna protection device. To compensate for the shortcomings of previous devices, it is necessary to develop a new protection device that has a short response time and high power tolerance; therefore, the development and research of plasma discharge-based devices is being conducted^30–33^. The design of the plasma limiter is based on the gas breakdown owing to the high input power. The plasma generated by the gas breakdown reflects the incident high-power signal. In general, plasma limiters have the advantage of operating well in the very-high-frequency band and under high input power. The plasma limiter should attenuate the transmitted power at the operating frequency to avoid damaging the receiver when a high-power EMP is injected. However, when inputting a weak signal, such as a normal signal, the insertion loss of the plasma limiter is minimized such that the sensitivity of the receiver is maintained. In general, the plasma limiter in waveguide technology has cone-shaped discharging point with or without inductive iris structure and has a disadvantage in that it is difficult to select an operating frequency and response to an uncertain incident power level. The resonator structure in the plasma limiter allows to reduce the power threshold, however, it results narrow operational bandwidth^31^. Among various approach, capacitive switching mechanism has been introduced to modulate the microwave based on the semiconductor device^34–36^. As the capacitance of the device is changed, the resonant frequency of the structure is changed. In this study, to improve the response power level and time of the plasma limiter and the insertion loss in the desired operating frequency band, a new structural design based on the resonance is proposed, and an appropriate inert gas pressure value is derived^37^.

## Results

### Design of the structure

**Figure 1:**
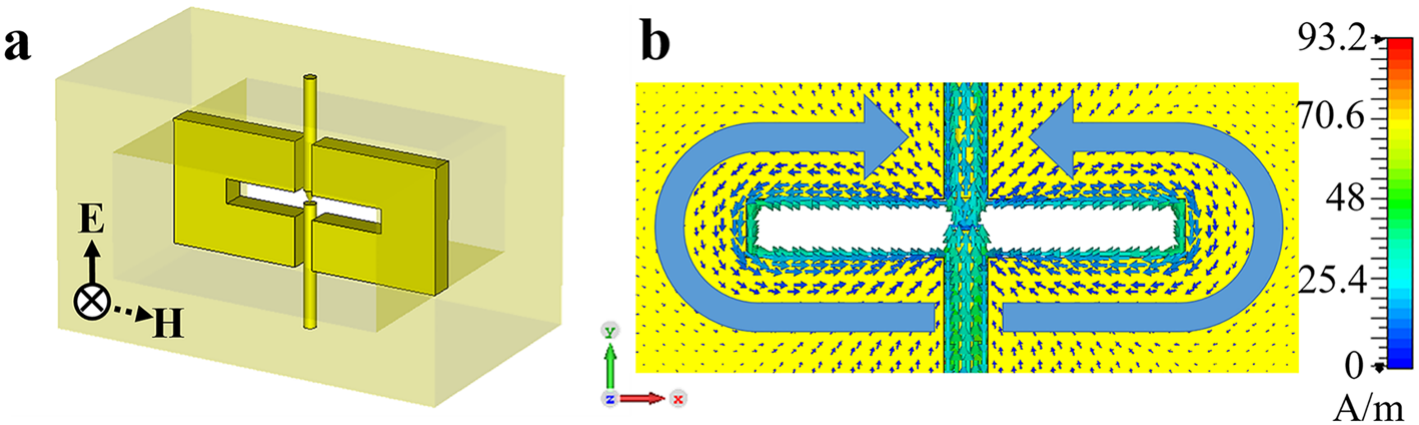
Design of the plasma limiter. (**a**) Schematic of a plasma limiter consisting of upper/lower electrodes and the slot structure. (**b**) Simulated surface-current (J_m_) distributions on the front-side slot structure.

A waveguide structure with selective-frequency band-pass characteristics and a shape that concentrates the electric field at a specific point is combined, as shown in Fig. 1a. Figure 1b presents the surface-current distribution with a resonant frequency of 9.4 GHz and the incident power of 1 W when the discharge electrode and structure are combined. The structure has a slot shape, and a gap exists between the slot structure and the electrode; when the slot resonates at a specific frequency, the surface current is concentrated at the center of the slot. The current is simultaneously concentrated on the discharge electrode located at the center at 93.2 A/m, while the electrode structure only exhibits 17.0 A/m (see Supplementary Fig. 1), and the electric field is concentrated between the upper and lower electrodes, thereby lowering the response power level of the limiter. The operating principle of the proposed waveguide-type plasma limiter is as follows. When a normal signal with low power passes through the plasma limiter, it is transmitted without an insertion loss. However, when a high-power EMP passes through the plasma limiter, the gas in the waveguide changes to a weakly ionized state, and the input signal is reflected without being transmitted. In addition, as described, the device that protects against the EMP threat has a short response time and must be designed to withstand a high power. This suggests that a design is required such that the transmission level changes significantly even in a small amount of plasma at a low input power, and the device operates stably.

**Figure 2:**
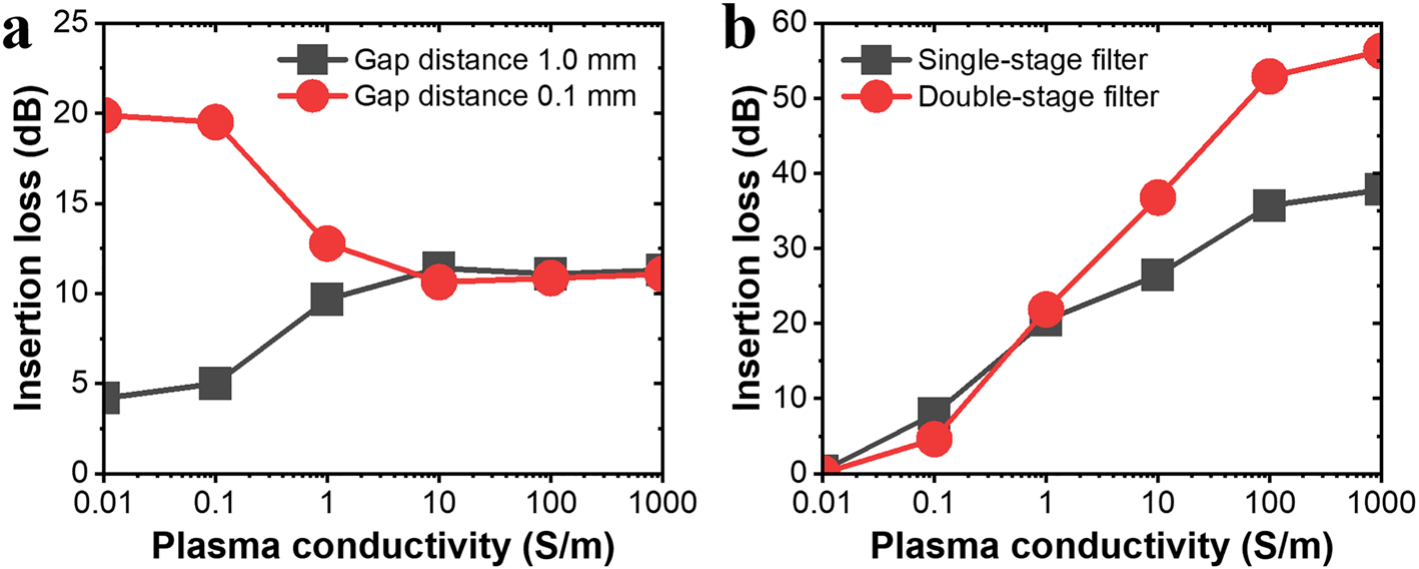
Numerical characterizations of the plasma limiters. (**a**) Insertion loss of needle-shaped electrode; (**b**) insertion loss of slot structure combined with electrode depending on cube-shaped plasma conductivity at the center between the upper and lower electrodes.

In the case of an EMP protection device, there are various methods of meeting the requirements; however, the most important factor is the discharge condition at which the gas should change to a weakly ionized state. Typically, a pointed needle is placed at the center of the waveguide to provide an appropriate separation distance^38–41^. A strong electric field is generated by inducing an applied EMP between the needles. At the target resonating frequency of 9.45 GHz, the needle-shaped structure and proposed slot structures are compared in Fig. 2. As the electrons in the microwave plasma follow the electromagnetic waves, the properties of plasma can be described by the Drude model^42^. The permittivity of plasma is consisted of real and imaginary parts. As the relative permittivity ${\varepsilon }_{r}=1$ for vacuum, the conductivity is considered as an imaginary part^43–45^. Frequency-dependent permittivity ${\varvec{\upvarepsilon}}({\varvec{\upomega}})$ can be derived as ${\varvec{\upvarepsilon}}\left({\varvec{\omega}}\right)=1-\left[{\varvec{i}}{\varvec{\upsigma}}\left({\varvec{\upomega}}\right)\right]/{\varvec{\upomega}}{{\varvec{\varepsilon}}}_{0}$ where an expression for the conductivity ${\varvec{\upsigma}}({\varvec{\upomega}})$ containing the electron density ${{\varvec{n}}}_{{\varvec{e}}}$, an electron charge ${\varvec{e}}$**,** with the mass ${{\varvec{m}}}_{{\varvec{e}}}$, and the collision frequency ${\varvec{\nu}}$ can be derived as ${\varvec{\upsigma}}({\varvec{\upomega}})=\left[{{\varvec{e}}}^{2}{{\varvec{n}}}_{{\varvec{e}}}\right]/[{{\varvec{m}}}_{{\varvec{e}}}({\varvec{\nu}}+{\varvec{i}}{\varvec{\omega}})]$. Assuming that there is a 2 cm × 2 cm × 2 cm local plasma at the center of the electrode^46^, the transmission was compared while changing the plasma conductivity, as shown in Fig. 2a. The needle-shaped plasma limiter interferes with the normal signal owing to high insertion loss even in the initial stage, that is, in the state of a plasma conductivity of 0.01 S/m. To reduce the insertion loss of needle-shaped plasma limiter, the gap distance between upper and lower electrode was increased from 0.1 to 1.0 mm. In addition, assuming that the plasma is generated by an EMP and the conductivity is increased, the insertion loss of 0.1 mm-gap-distance needle-shaped plasma limiter is reduced in the initial generation process when the conductivity of the plasma is 10 S/m or less, indicating a vulnerable state. In the case of a plasma conductivity of 10 S/m or higher, the insertion loss exhibited an attenuation performance of 11 dB regardless of gap distance between electrodes. In the case of the proposed slot structure plasma limiter with 0.1 mm-gap-distance, as shown in Fig. 2b, on assuming that plasma generation is in the initial state, when the plasma conductivity is 0.01 S/m, the insertion loss is lower than 0.5 dB for both single-stage and double-stage slot structure, and thus, it transmits the normal signal well without interference. It can be observed that the insertion loss was 20 dB even in the initial generation process when plasma was generated, and the plasma conductivity was increased to 1 S/m, thus exhibiting a higher blocking coefficient compared to the 11 dB performance of the needle-shaped plasma limiter. As the plasma conductivity increased, the incident power increased, and both structures exhibited a higher blocking coefficient. Compared with the needle-shaped plasma limiter, the proposed slot structure design exhibits a higher electric-field concentration and blocking coefficient for an incident signal. Thus, a difference in the response time and response power level of the plasma discharge according to the difference in the electric field strength is expected.

**Figure 3:**
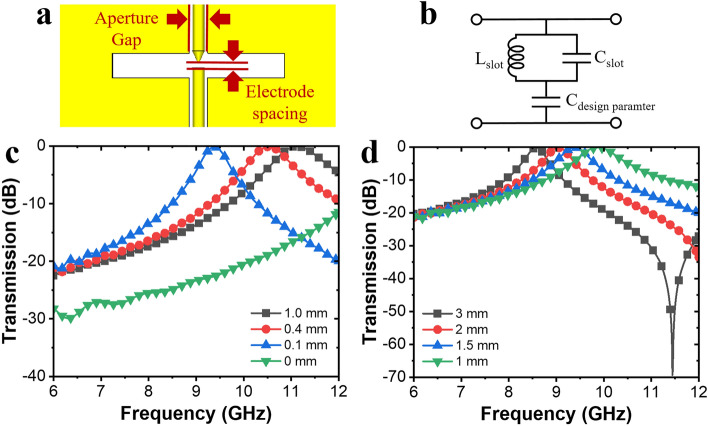
Design parameter optimization for the plasma limiters. Schematic of (**a**) electrode spacing and gap variation between the electrode and slot structures. (**b**) Equivalent circuit of proposed design. Simulated transmission spectra of variation in (**c**) electrode spacing and (**d**) aperture gap.

As shown in Fig. 3a, the transmission characteristics change according to the distance between the electrodes in the slot structure were simulated. The electrode spacing was varied in micrometers. The proposed slot structure based on LC resonance is shown in Fig. 3b. Owing to the variation in electrode spacing, a change in the resonant frequency was observed, as shown in Fig. 3c. As a fabrication-process defect exists, the spacing variation can be utilized to compensate for the error, and the resonant frequency is adjusted to the target point. In the case of electrodes connected to each other with a spacing of 0 mm, the resonance was weaker owing to destructive interference between the surface-current paths. The electric field strength changes in free space according to the electrode spacing (see Supplementary Fig. 2). The smaller the distance between the electrodes, the greater the electric field strength, and the frequency shifts to a lower band. While maintaining the shortest distance of electrode spacing, aperture gap is required to adjust the vertical position of the electrodes. The electrode spacing was fixed at 100 μm with a resonant frequency of 9.45 GHz, and the effects of the electrode entering the gap between the apertures were compared, as shown in Fig. 3d. The resonant frequency shifted to a higher frequency band as the gap between the electrodes decreased.

**Figure 4:**
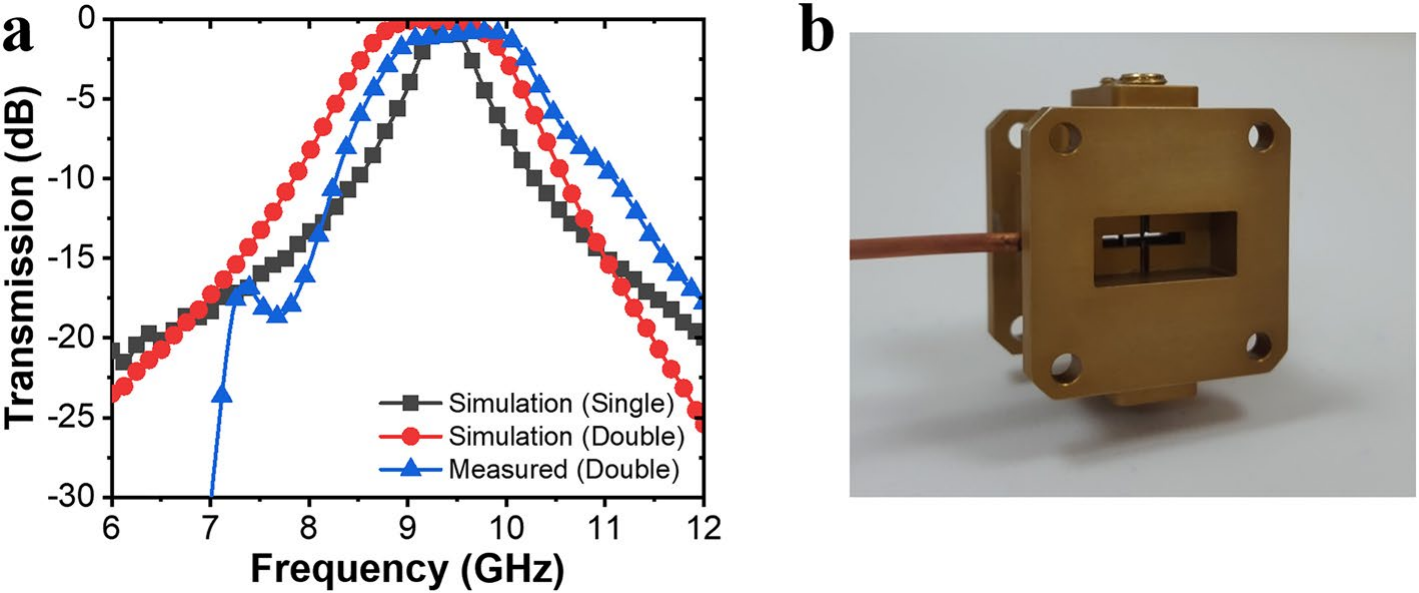
Comparison of simulated and measured transmission characteristics of a plasma limiter. (**a**) Transmission spectra of the simulated and experimental results of a plasma limiter. (**b**) Photograph of demonstrated plasma limiter.

Figure 4 shows the transfer characteristics of the double-stage slot structure used in this study. When inputting a normal signal, the insertion loss of the plasma limiter has to be minimized. When a single-stage slot structure is used, a low insertion loss is observed in a narrow band, but when a double-stage slot structure is used, a low insertion loss is observed in a wider band acting as a coupled resonator structure. The insertion loss, which is a transmission characteristic, is minimized by optimizing the proposed slot structure. The designed double-stage slot structure exhibited an insertion loss of 0.13 dB at 9.45 GHz and < 0.5 dB in the 8.83–9.68-GHz band. For the optimization, the parameters of each detailed structure were adjusted in millimeters (see “Methods” section). On referring to the plasma limiter actually used, the total waveguide length was limited to within 2 cm, and the device was made compact while maintaining various performance indexes such that the end-user could install it in the desired place without any restrictions, as shown in Fig. 4b. The transmission characteristics of the double-stage slot structure were measured using a network analyzer. The insertion loss was 1.01 dB at 9.45 GHz and 0.78 dB at 9.75 GHz. In the 9.35–10.00-GHz frequency regime, the insertion loss was less than 1.10 dB. After the fabrication, the additional insertion loss owing to the process error and dielectric constant of the vacuum window increased.

### Appropriate gas pressure

**Figure 5:**
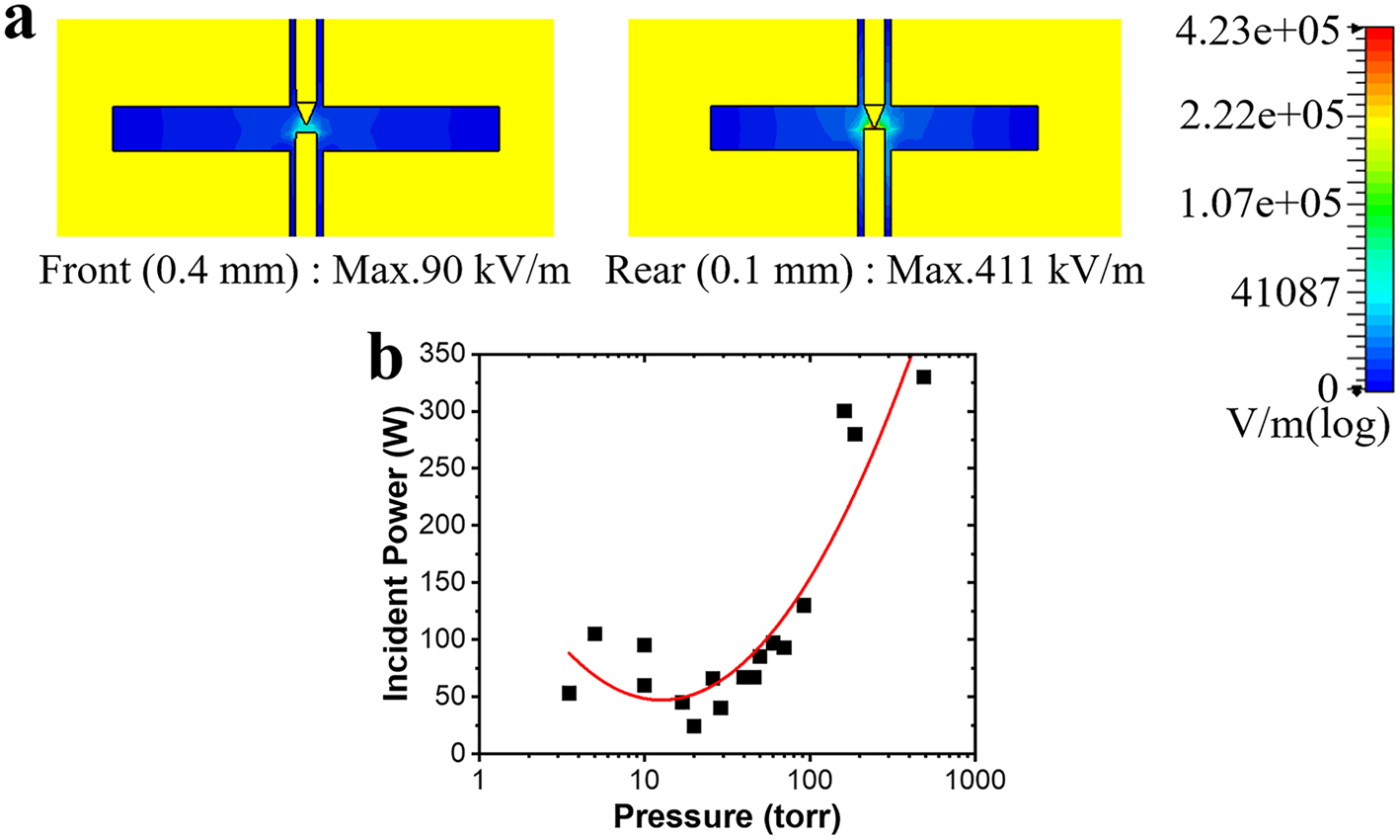
Xenon-gas pressure optimization. (**a**) Electric-field distribution with a resonant frequency of 9.45 GHz and the incident power of 1 W in a free space with a double-stage slot structure. (**b**) Incident power graph at the point when the gas breakdown occurs; Paschen’s curve.

Figure 5a shows the value of the electric field in the free space between the electrodes at the front and rear ends of the double-stage slot structure. An electric field is concentrated between the electrodes, and the mean kinetic energy of electrons is larger than that of the gas temperature thus causing gas breakdown to form the non-equilibrium plasma state^47^. The upper and lower electrodes had a conical and cylindrical tip, respectively. A gas breakdown occurred between the upper and lower electrodes, and a plasma discharge was generated. As the second-stage slot structure has a shorter electrode distance of 100 µm, the electric field is larger than that in the first-stage slot structure. The plasma discharge begins at the second- and first-stage slot structures in a sequence when the EMP signal interferes with the band-pass frequency regime. The inert gas was selected based on previous reports^38,39,48,49^. Among argon (100%), argon (0.6%)/neon (99.4%), xenon (1.6%)/neon (98.4%), and xenon (100%), the pure xenon gas exhibited the most stable and fastest response. The photo of plasma discharge is given in Supplementary Fig. 3. And the plasma sheaths can be observed in Supplementary Fig. 3(a). The additional parasitic capacitances based on plasma sheaths will shift the resonant frequency to a higher frequency band^50^. Because the gas discharge condition depends on both the gas pressure and electrode distance, Paschen’s curve was extracted, as shown in Fig. 5b. The plasma discharge was conducted at inside of metallic waveguide with constant gas pressure at room temperature, while maintaining the pressure using a vacuum window on both sides of the plasma limiter. While maintaining the shortest gap distance of 100 μm, gas breakdown occurs at 20 Torr at the lowest 30 W incident power.

### High-power injections

**Figure 6:**
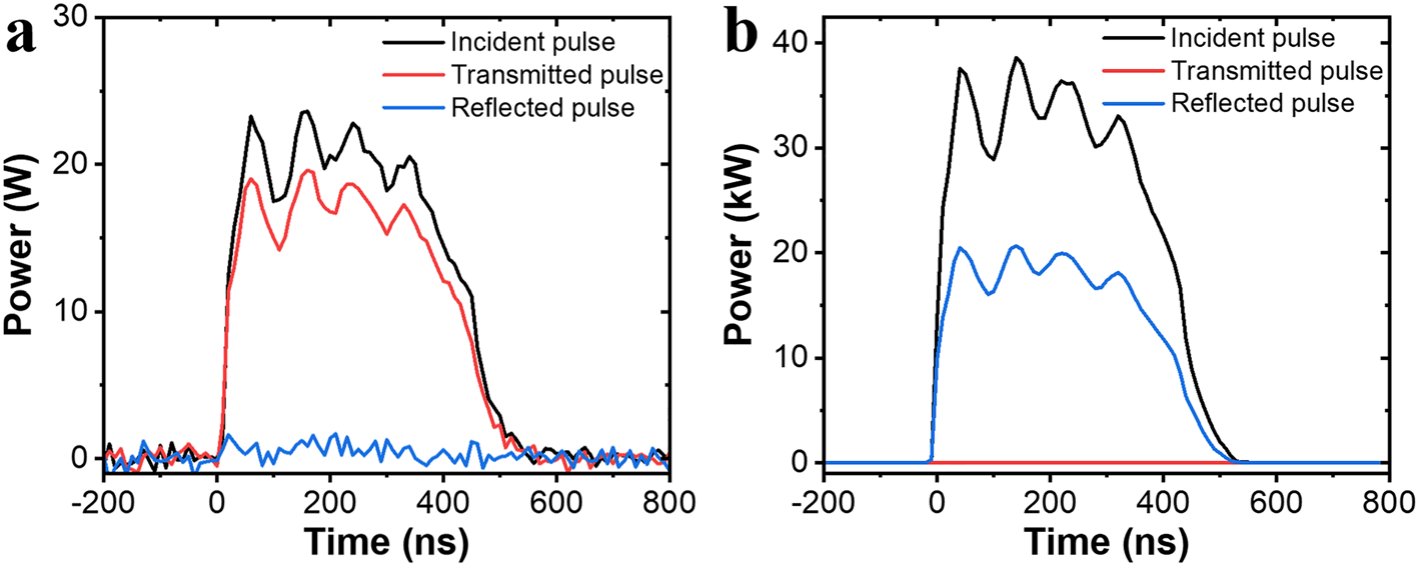
Measurements of the plasma limiter in the time domain. Incident, transmitted, and reflected pulses of plasma limiter for a (**a**) normal signal with an incident power level of 25 W, (**b**) high-power signal with an incident power level of 40 kW.

Figure 6 presents the transmission characteristics for the normal and operating conditions of the demonstrated plasma limiter in the time domain. The corresponding characteristic is the result of the measurement in the plasma limiter waveguide test bed, and the transfer characteristics were measured in real-time using a power meter (see “Methods” section). In Fig. 6a, assuming that the low input power is a normal signal, the transmitted pulse shows the same pulse waveform with insertion loss as the pulse generated from the source. In the normal state, with a smaller difference between the input and transmitted pulses, the insertion loss is lower. The reflected waveform exhibits a characteristic convergence to almost zero. In Fig. 6b, it was assumed that the high input power was a transient or EMP signal, and the transmitted pulse shows a transmission characteristic that converges to almost zero, with a blocking efficiency of 40.55 dB. The reflected power level was about half of the input power and the rest of input power was absorbed into thermal energy. In addition, the leakage power was around 2 W after gas breakdown and the duration of the transmission waveform was less than that at 10 ns, which demonstrated the response time characteristics. And the recovery time was less than 400 ns as shown in Supplementary Fig. 4. Both response and recovery time characteristics were stable regardless of the incident power level.

## Discussion

In summary, the slot structure and discharge electrode were combined and exhibited band-pass characteristics at 9.45 GHz. When a normal signal is injected, a transmission close to 1 exhibited ideal characteristics. When a high-power EMP is applied from the outside, plasma is generated between the electrodes, and the characteristic change can be confirmed as the conductivity of the plasma changes. The state wherein the conductivity of the plasma was 1 S/m exhibited reflective properties in all the frequency bands, and the properties improved as the conductivity increased. In general, plasma in a weakly ionized state is reported to have 1000 S/m^51^, and it is expected that the proposed design has a lower response-power level in the initial stage, as it has the desired characteristics even at 1 S/m, which is the 1/1000 level. In the case of high-power EMP is applied, the blocking efficiency of 40.55 dB was observed in Fig. 6b and the plasma conductivity can be concluded around 10 S/m from Fig. 2b. Comparing with the single-stage slot structure, the double-stage slot structure exhibited wide-band transmission while maintaining a low insertion loss acting as a coupled resonator structure. Outside the operating frequency band, the EMP will be reflected through the band pass filter. In the time domain, the plasma discharge was generated within 10 ns by the high-power EMP, and the majority of the incident power was reflected. The gas breakdown occurs at 30 W incident power which can be a high level to fully protect a receiver. The diode limiter can be connected behind the plasma limiter to suppress the leakage power. The demonstrated plasma limiter is useful for the operation of the RADAR system to protect the receiver. As a constant-communication environment is required, stable operation, even with the EMP threat and lightning conditions, would help the end-user.

## Methods

### Device fabrication

The dip-brazing method was used for the production of waveguide-type plasma limiters. The external parts were connected by soldering, and an O-ring was placed in the device to adjust the spacing of the center electrode to maintain a vacuum. Supplementary Fig. 5 shows the main design parameters of the proposed plasma limiter. Parameters A and B affect the determination of the operating frequency, while parameters C, D, and E affect the insertion loss and electric-field concentration. The values of the plasma limiter were determined based on the final schematic presented in Supplementary Fig. 5b. The plasma limiter was designed to comply with the international standard WR-90 and designed using SI units for convenience of the manufacturing process.

### Numerical simulation

Electromagnetic simulations were performed using a commercial software (CST). Within the waveguide model, the plane wave was placed on the front side, and the probe was located on the opposite side to detect the electromagnetic response of the devices. The boundary conditions of a perfect electrical conductor were imposed at the top and bottom, and the boundary conditions of a perfect magnetic conductor were imposed on the left and right.

### Plasma limiter test bed

A 40-kW peak-power X-band pulse magnetron was installed for use as an EMP source. The waveguide-type variable attenuator, front-side directional coupler, plasma limiter, back-side directional coupler, and terminator were connected in sequence, as shown in Supplementary Fig. 6. The directional couplers were connected to a power meter to measure the incident, reflected, and transmitted pulse signals in the time domain. The plasma limiter had a gas pumping line in the sidewall and was connected to a vacuum pump, vacuum gauge, and xenon gas cylinder to control the gas pressure. The gas pressure was managed as a static gas. The cavity was pumped to a very low pressure, then it filled with a gas at the correct pressure, and finally close a valve of the device and turn off the pumping system.

## Supplementary Information

Supplementary Information.

## Data Availability

The datasets used and/or analyzed during the current study available from the corresponding author on reasonable request.
