# Supplementary material for: Plasma-discharge-integrated slot structure for microwave power limiter
Source: Sci Rep. 2023 Jun 22;13:10156. doi: 10.1038/s41598-023-37336-1 (PMC10287632; doi:10.1038/s41598-023-37336-1)
Supplement: Supplementary file 1 — Supplementary Information. [file 41598_2023_37336_MOESM1_ESM.docx]

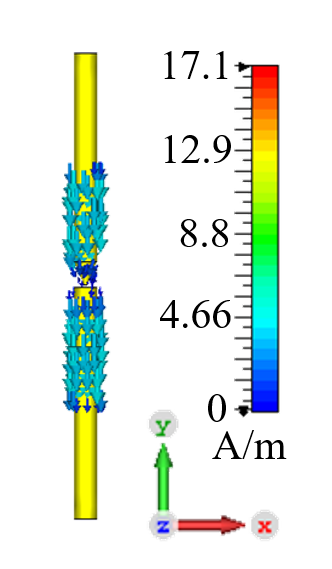


**Supplementary Figure 1| Simulation of the needle-shaped electrode.**

Simulated surface-current (J_m_) distributions on the electrode-only structure.

**
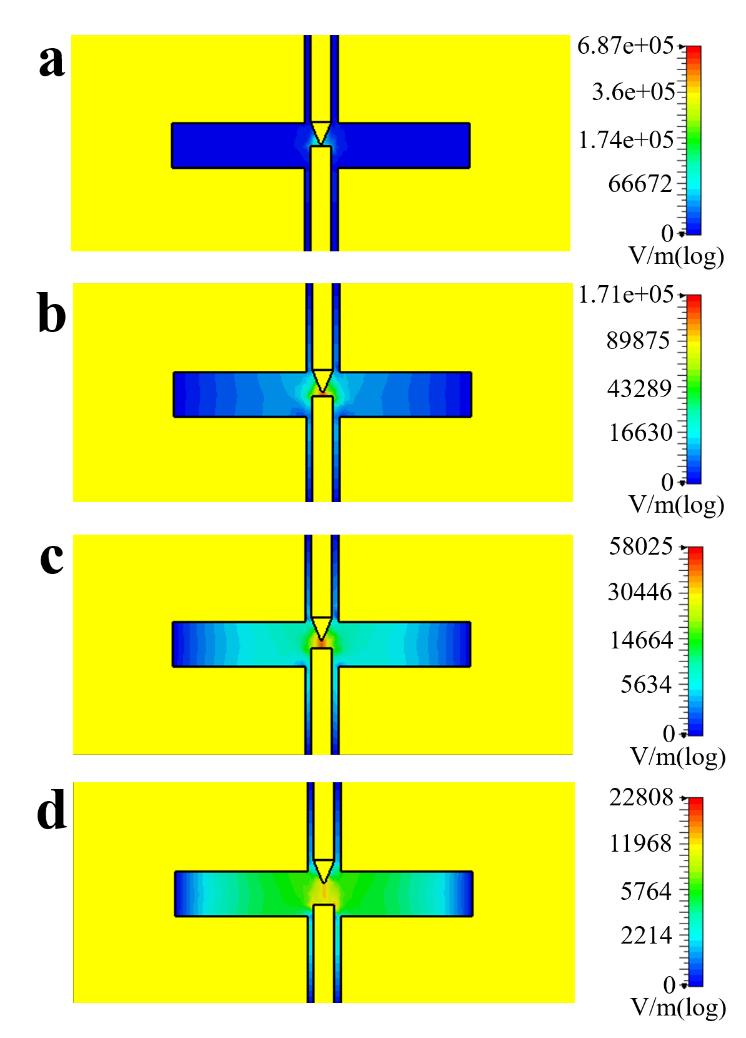
**

**Supplementary Figure 2| Spatial electric-field distribution of the slot structure plasma limiter.** Electrode-spacing variations were as follows: **(a)** 0 mm, **(b)** 0.1 mm, **(c)** 0.4 mm, and **(d)** 1.0 mm.

**
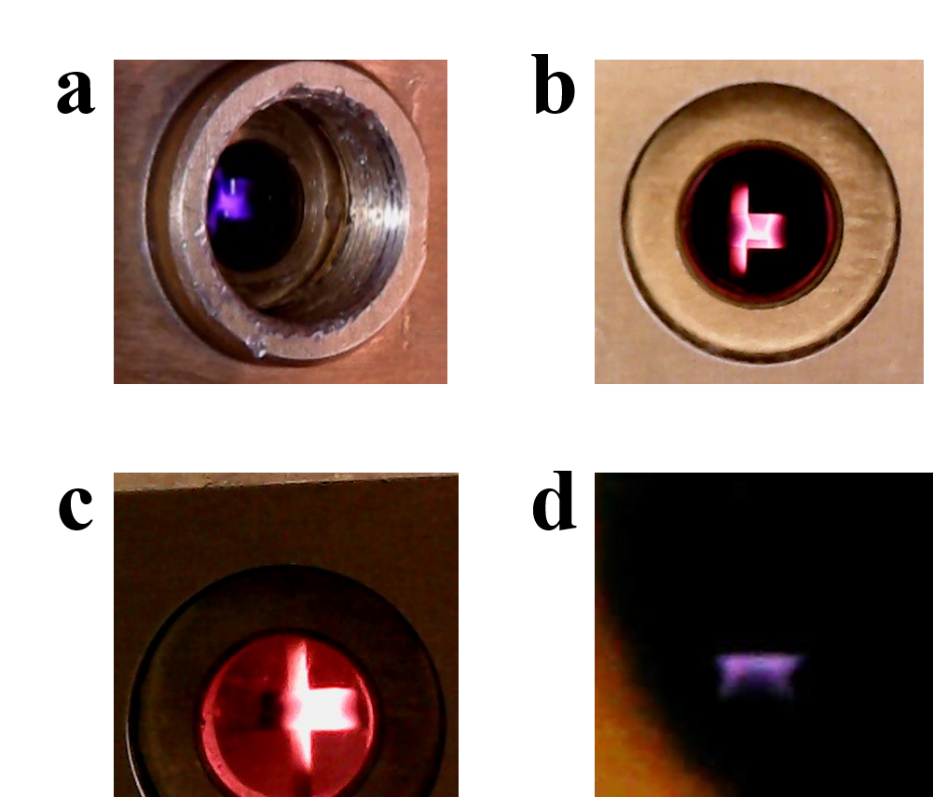
**

**Supplementary Figure 3| Photograph of plasma discharge**

The vacuum viewport included plasma limiter was demonstrated to capture the plasma discharge. The composition of the gas is given as follows. **(a)** argon 100% **(b)** argon (0.6%)/neon (99.4%) **(c)** xenon (1.6%)/neon (98.4%) **(d)** xenon (100%)


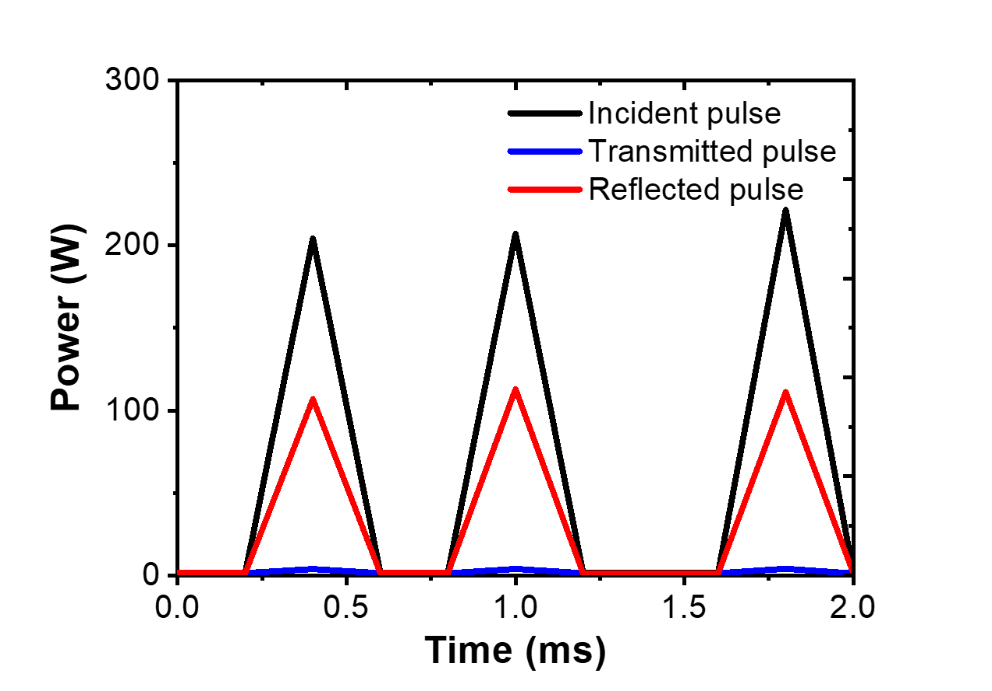


**Supplementary Figure 4| Measurements of the plasma limiter in the time domain.**

The plasma limiter measurement period was 200 ns and the incident pulse period was less than 600 ns.


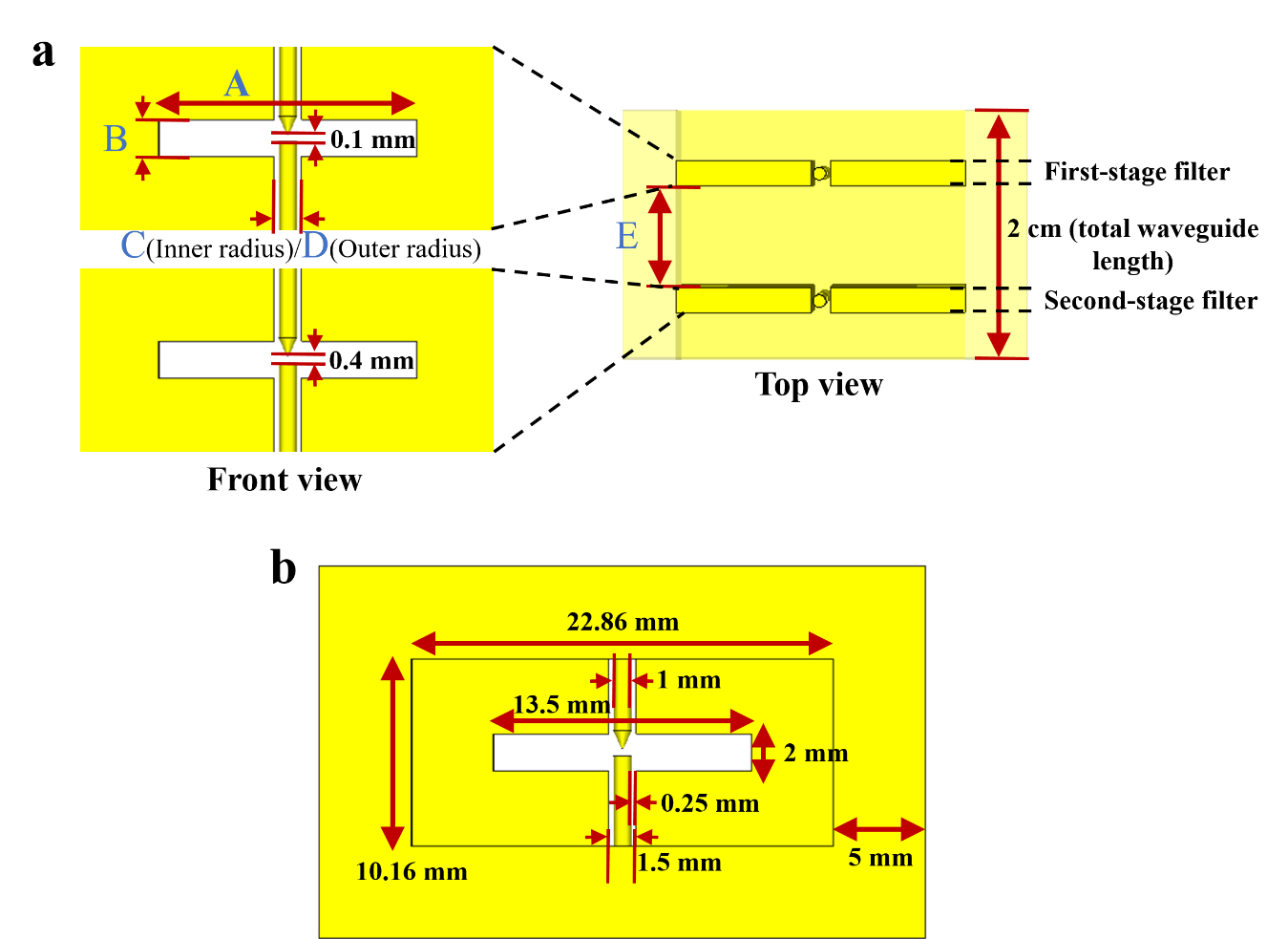


**Supplementary Figure 5| Design-parameter optimization of the slot structure plasma limiters.**

**(a)** Front and top views of double-stage slot structure with the following geometric parameters: A: Slot length (13.5 mm), B: Slot width (2 mm), C: Inner radius (1 mm), D: Outer radius (1.5 mm), E: Distance between first and second stage filter (8 mm) **(b)** Optimized parameters.


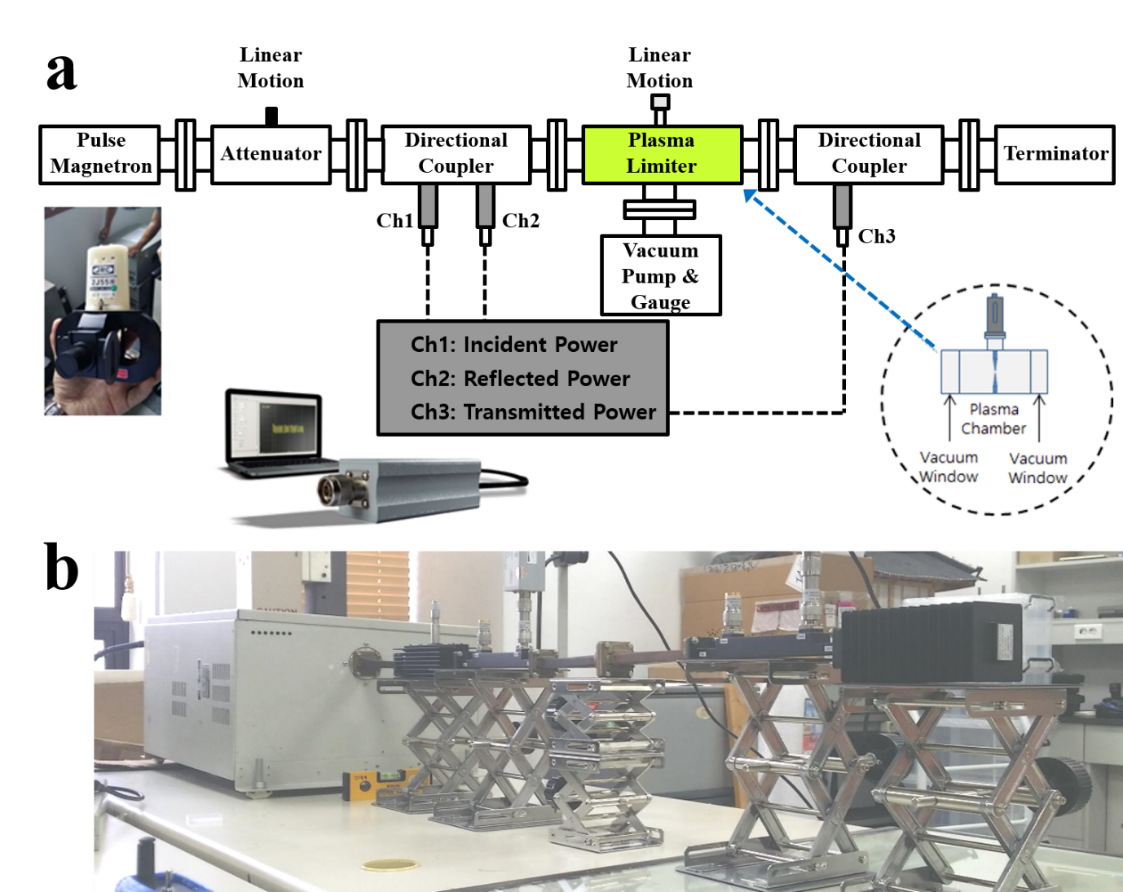


**Supplementary Figure 6| Measurements of the plasma limiter test bed in the time domain. (a)** Schematic of plasma limiter test bed. **(b)** Photograph of plasma limiter test bed.
